# Requirement of Cognate CD4^+^ T-Cell Recognition for the Regulation of Allospecific CTL by Human CD4^+^CD127^−^CD25^+^FOXP3^+^ Cells Generated in MLR

**DOI:** 10.1371/journal.pone.0022450

**Published:** 2011-07-22

**Authors:** Yuming Yu, Joshua Miller, Joseph R. Leventhal, Anat R. Tambur, Dhivya Chandrasekaran, Josh Levitsky, Xunrong Luo, James M. Mathew

**Affiliations:** 1 Department of Surgery, Comprehensive Transplant Center, Northwestern University Feinberg School of Medicine, Chicago, Illinois, United States of America; 2 Department of Organ Transplantation, Nanfang Hospital, Southern Medical University, Guangzhou, China; 3 Jesse Brown VA Medical Center, Chicago, Illinois, United States of America; 4 Division of Hepatology, Department of Medicine, Northwestern University Feinberg School of Medicine, Chicago, Illinois, United States of America; 5 Division of Nephrology and Hypertension, Department of Medicine, Northwestern University Feinberg School of Medicine, Chicago, Illinois, United States of America; 6 Department of Microbiology-Immunology, Northwestern University Feinberg School of Medicine, Chicago, Illinois, United States of America; Blood Systems Research Institute, United States of America

## Abstract

Although immunoregulation of alloreactive human CTLs has been described, the direct influence of CD4^+^ Tregs on CD8^+^ cytotoxicity and the interactive mechanisms have not been well clarified. Therefore, human CD4^+^CD127^−^CD25^+^FOXP3^+^ Tregs were generated in MLR, immunoselected and their allospecific regulatory functions and associated mechanisms were then tested using modified ^51^Chromium release assays (Micro-CML), MLRs and CFSE-based multi-fluorochrome flow cytometry proliferation assays. It was observed that increased numbers of CD4^+^CD127^−^CD25^+^FOXP3^+^ cells were generated after a 7 day MLR. After immunoselection for CD4^+^CD127^−^CD25^+^ cells, they were designated as MLR-Tregs. When added as third component modulators, MLR-Tregs inhibited the alloreactive proliferation of autologous PBMC in a concentration dependent manner. The inhibition was quasi-antigen specific, in that the inhibition was non-specific at higher MLR-Treg modulator doses, but non-specificity disappeared with lower numbers at which specific inhibition was still significant. When tested in micro-CML assays CTL inhibition occurred with PBMC and purified CD8^+^ responders. However, antigen specificity of CTL inhibition was observed only with unpurified PBMC responders and not with purified CD8^+^ responders or even with CD8^+^ responders plus Non-T “APC”. However, allospecificity of CTL regulation was restored when autologous purified CD4^+^ T cells were added to the CD8^+^ responders. Proliferation of CD8^+^ cells was suppressed by MLR-Tregs in the presence or absence of IL-2. Inhibition by MLR-Tregs was mediated through down-regulation of intracellular perforin, granzyme B and membrane-bound CD25 molecules on the responding CD8^+^ cells. Therefore, it was concluded that human CD4^+^CD127^−^CD25^+^FOXP3^+^ MLR-Tregs down-regulate alloreactive cytotoxic responses. Regulatory allospecificity, however, requires the presence of cognate responding CD4^+^ T cells. CD8^+^ CTL regulatory mechanisms include impaired proliferation, reduced expression of cytolytic molecules and CD25^+^ activation epitopes.

## Introduction

CD4^+^ regulatory T cells (Tregs) are proposed to play a key role in the generation and maintenance of tolerance to organ and tissue allotransplants [1], [2],[3]. Experiments in rodent models have shown regulatory effects on cytotoxic T cells (CTLs) by CD4^+^ Tregs [4], [5]. In humans, CD4^+^ Tregs have been demonstrated to impair CTL function in the settings of cancer [6], and chronic viral diseases [7], [8], [9], [10]. CD8^+^ cytotoxic T lymphocytes (CTLs) can also be demonstrated post-transplantation even in patients who have stable graft function [11], [12], [13], possibly implying regulatory control. Although regulation of CD8^+^ T cells has also been described in alloimmunity [14], the direct influence of human CD4^+^ Tregs on CD8^+^ cytotoxicity and the mechanisms of this interaction have not been well clarified. In human renal allograft biopsies in acute rejection in which putatively regulatory Forkhead/winged-helix protein 3 (FOXP3) staining cells have predominated clinically favorable prognoses have been reported [15]. Similar findings have been described in the urine “compartment” in such recipients [16].

Since many of the findings in animal models are not applicable in humans and since many experiments cannot be performed in the human, we have used *ex vivo* culture systems to analyze the role of regulatory T cells on alloimmunity. We have previously reported that increased numbers of human CD4^+^CD127^−^CD25^+^FOXP3^+^ cells are generated after a 7 day bulk mixed lymphocyte reaction (MLR) and that when isolated (MLR-Tregs) and added as third components, these cells allospecifically inhibited a primary MLR as well as caused increased percentages of newly generated CD4^+^CD127^−^CD25^+^FOXP3^+^ T cells termed “regulation recruitment” [17]. In a clinical tolerance study, we have observed that the percentages of CD4^+^CD127^−^CD25^high^FOXP3^+^ cells increased by 10-fold from the pre-operative values during the first 6 months and remained >4-fold even after 24 months in the peripheral blood mononuclear cells (PBMC) of Human leukocyte antigen (HLA) -identical kidney recipients. This protocol involved alemtuzumab induction, donor CD34^+^ hematopoietic stem cell infusion, and Tacrolimus to Sirolimus conversion followed by slow withdrawal of immunosuppression [18]. In this study, when post-op recipient PBMC containing these high percentages of putative Tregs were added as third component modulators, they inhibited the donor-specific proliferation of cryopreserved pre-op recipient CFSE-labeled PBMC responders, as well as enhanced the newly generated CD4^+^CD127^−^CD25^high^FOXP3^+^ cells in the CFSE labeled proliferating responders [17], [18]. In the present report, e*x vivo* generated MLR-Tregs have been tested as modulator cells for their effects in a modified Cell Mediated Lympholysis (micro-CML) ^51^Chromium release assay to measure CTL regulation. It was questioned whether these MLR-Tregs could regulate the generation and cytotoxicity of CD8^+^ CTL and whether this regulation had allospecificity. Additional mechanisms of the CD4^+^/CTL regulatory effect were probed by experiments measuring MLR-Treg effects on CD8^+^ proliferation, and the expression of cytolytic, apoptotic and activation molecules.

## Materials and Methods

### Human subjects and HLA Typing

Peripheral blood mononuclear cells (PBMC) were obtained from healthy volunteers that were HLA typed by the Northwestern histocompatibility laboratory using molecular methods. They were selected for this study to be HLA- A, B and DR mismatched with each other. The research was conducted on these human subjects with the approval of the Northwestern Institutional Review Board. Informed written consent was obtained from each human subject.

### Generation of Tregs in MLR

**Figure 1:**
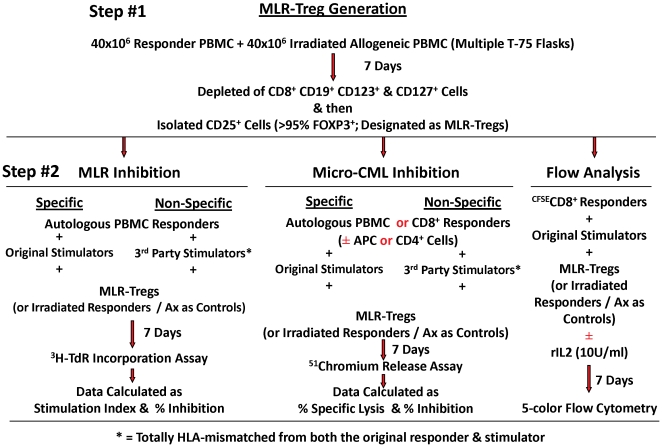
Flow diagram depicting the culture system for the generation of MLR-Tregs (step #1) and their utilization in various MLR, micro- CML and flow cytometric assays (step #2).

MLR-Tregs were generated as we previously reported [17] and as shown in the top portion of Figure 1. Briefly, PBMC were isolated by Ficoll-Hypaque density gradient centrifugation and 40×10^6^ responder cells were stimulated with 40×10^6^ irradiated (3000 R) stimulator cells in culture medium [NAB-CM; RPMI-1640 supplemented with 2 mM L-glutamine, 10 mM HEPES, 100 U/ml Penicillin-Streptomycin (all from Mediatech, Manassas, VA) and 15% normal human AB serum (Gemini Bio-Products, W. Sacramento, CA)] at 1×10^6^ cells/ml at 37°C in 5% CO_2_ in multiple T-75 flasks. After 7 days, the CD4^+^CD127^−^CD25^+^ cells were purified using the Treg isolation kit and the AutoMACS (Miltenyi Biotech, Auburn, CA) as previously described [17].

### Immunophenotyping of MLR-Tregs

As previously described [19], immunophenotyping for surface markers CD3, CD4, CD8, CD25 and CD127 was performed with monoclonal antibodies directly conjugated with one of four fluorochromes, that is, fluorescein isothiocyanate (FITC), phycoerythrin (PE), PE-cyanin 5 (PC5), and PE-cyanin 7 (PC7) (Beckman-Coulter, Miami, FL). Intracellular FOXP3 staining was performed using PE-conjugated FOXP3 kits (eBiosciences, San Diego, CA) following the manufacturer's instructions. Readings were performed in a 5-color FC500 flow cytometer (Beckman Coulter), by analysis for 1×10^5^ cellular events. Isotype controls were used to determine background fluorescence.

### Suppression of MLR proliferation by MLR-Tregs

MLR-Tregs or autologous responder irradiated PBMC controls were added as modulators (10,000, 2,000 and 400) to freshly prepared MLRs in triplicates containing 1×10^5^ responder and stimulator PBMC in 96-well U-bottom plates. These readout MLRs for donor-specific suppression contained cells obtained from the original fully HLA mismatched responder/stimulator combination used in generating the MLR-Tregs. However, for assessment of non-specific suppression in the readout MLR, the irradiated stimulator PBMC used were from a different and fully HLA mismatched (third party) individual than the one used in generating the MLR-Tregs. After 7 days in culture, 1 uCi ^3^H-TdR was added for 18 hrs and the cultures were harvested. Radioactive incorporation was measured as CPM in a Perkin-Elmer scintillation counter. The percentage of inhibition by the Tregs was calculated using the formula: [1−(CPM in presence of Treg modulators) / (CPM in presence of control modulators)×100].

### Cell subset isolation from PBMC

Purified CD8^+^ T cells and CD4^+^ T cells were isolated from fresh PBMCs using CD8 microbeads or CD4 microbeads (Miltenyi Biotech) respectively by positive selection according to the manufacturer's protocol. The purity was over 99% as estimated by flow cytometric analysis. Non-T antigen presenting cells (APCs) were isolated by depletion of CD2^+^ cells from fresh PBMCs by CD2 microbeads (Miltenyi Biotech) according to the manufacturer's protocol. These were an admixture of cells with a purity of non-T cells of over 96% as assessed by flow cytometric analysis.

### Micro-Cell-mediated lympholysis (Micro-CML) [20]

Briefly, 1×10^5^ PBMC, 5×10^4^ purified CD8, 5×10^4^ purified CD8 plus non-T “APCs” or 5×10^4^ purified CD8 plus purified CD4^+^ responder cells were respectively stimulated with 1×10^5^ irradiated PBMCs in mixed culture replicates of 10 in 96-well, U-bottom plates at 0.2 ml/well in the absence or presence of 10 U/ml recombinant interleukin-2. The proportion of CD4^+^ or non-T “APC” added to the purified CD8^+^ responders were equivalent to that observed in the total PBMC of the individual blood donor. After a 7 day culture period, ^51^chromium-labeled PHA blast target cells (5×10^3^) were added to each mixed culture well. Four hours later, 25 µl supernatants without cells were transferred to a Lumaplate (Perkin-Elmer) and radioactivity was measured on a TopCount (Perkin-Elmer). Cultures with stimulator cells plus medium (i.e., no responder cells) served as negative controls (NC). Spontaneous and maximum release (SR and MR) were determined by adding target cells to wells containing NAB-CM or 1% Triton X-100, respectively. The data were expressed as follows: % specific lysis = [(Mean CPM in sample)−(Mean CPM in NC) / (Mean CPM in MR)−(Mean CPM in SR)]×100. For assessing suppression, MLR-Tregs or irradiated autologous PBMC controls were added as modulators at dilutions of 10,000, 2,000 or 400 cells to the micro-CML wells at the time of mixed culture preparation on day 0 (Figure 1). The percentage of inhibition of lysis by the MLR-Tregs was calculated using the formula: [1−(% Specific Lysis in presence of Treg modulators) / (% Specific Lysis in presence of control modulators)×100].

### CFSE or PKH26 staining of responder cells

Responder PBMC or purified CD8^+^ cells were labeled with the green fluorescent dye carboxyfluorescein diacetate, succinimidyl ester (CFSE) or red fluorescent dye PKH26 (both from Sigma-Aldrich, St. Louis, MO), as per the manufacturer's protocols. The efficiency of labeling was determined before the cells were used in experiments and was >95%. These labeled cells were used for assessing the regulatory functions of MLR-Tregs in flow cytometric analyses.

### Flow Cytometry Analyses of purified CD8 cells regulated by MLR-Tregs

10,000 MLR-Tregs or irradiated PBMC controls were added as modulators to 5×10^4^ purified CD8 (CFSE or PKH26 labeled) responders stimulated with 1×10^5^ irradiated PBMCs in 96-well, U-bottom plates at 0.2 ml/well in the absence or presence of 10 U/ml recombinant interleukin-2. After 7 days in culture, the cells were harvested, like cultures combined and 4-color flow cytometry was performed as above for cell surface expression with anti-human CD8-ECD, anti-human CD28-PC5, anti-human CD25-PC7 (all from Beckman-Coulter, Miami, FL) and anti-human FasL-FITC. Intracellular expression with anti-human perforin-FITC and anti-human granzyme B-FITC, (all from eBiosciences, San Diego, CA) was also measured in multiple tubes as per the manufacturer's instructions. In experiments where FITC-conjugated antibodies were used, the CD8^+^ responder cells were labeled with PKH26. Isotype controls were used to determine background fluorescence. The data were acquired for 100,000 events in a 5-color FC500 flow cytometer and analyzed using the CXP program (Beckman-Coulter).

### Statistical Methods

Data were depicted as means ± SD. Comparisons were performed by using the paired Student t-test. Differences were considered significant if P values were less than 0.05.

## Results

### Purity of CD4^+^CD127^−^CD25^+^ T Cells generated in MLR

**Figure 2:**
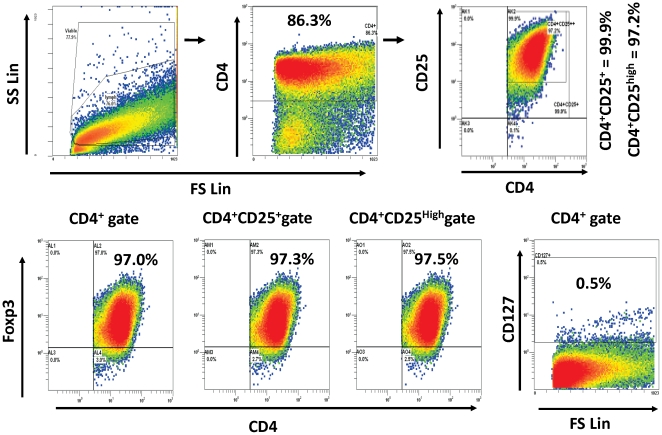
Purity and FOXP3 expression of MLR-Tregs. PBMCs from a healthy volunteer were stimulated with irradiated PBMC from an HLA fully mismatched donor. After 7 days, the CD4^+^CD127^−^CD25^+^ cells were isolated by the Treg isolation kit (methods). These cells were designated as MLR-Tregs. The purity of the isolated cells was assessed for the indicated markers by flow cytometry. The gating strategy is indicated by arrows and title headings on histograms. Thus, the viable cells (top left) were gated based on forward scatter and side scatter and then on CD4^+^ cells (top middle) followed by CD25^+^ or CD25^high^ expression (top right). The FOXP3 and CD127 levels were then assessed on indicated gates (bottom). These dot plots depict 1 example of >30 experiments performed in this report.

When responding PBMC were cultured for 7 days with irradiated HLA-A, B, DR mismatched stimulating PBMC, the generated CD4^+^CD127^−^CD25^+^ cells could be immunoselected (Methods and step 1 of Figure 1) to be >90% CD4^+^CD25^+^ and >99% CD127dim/^−^. Of these >95% were FOXP3^+^ (Figure 2). The preparations were designated as MLR-Tregs. These were added as third component modulators as described in the Methods section and in the lower portions (Part #2) of the flow diagram in Figure 1.

### MLR-Tregs suppressed MLR proliferation with allospecificity

**Figure 3:**
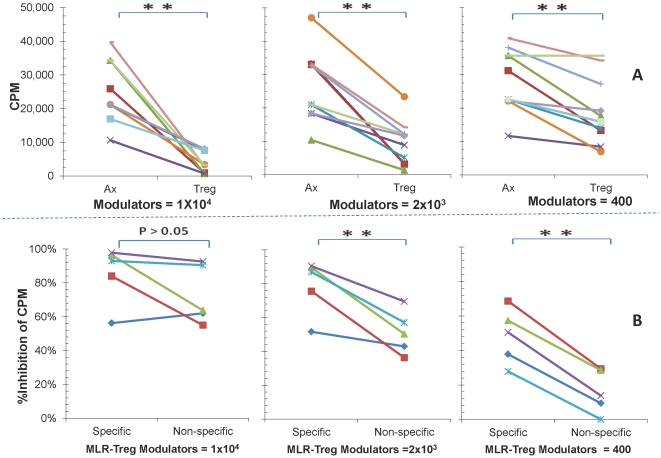
The ability of MLR-Tregs to allospecifically suppress MLR proliferation. MLR-Tregs were added as modulators in descending concentrations of 1×10^4^, 2×10^3^ or 0.4×10^3^ cells per well to 1×10^5^ fresh responding PBMC from the same individual as the one from whom MLR-Tregs were generated (i.e., the responders were autologous to MLR-Tregs). These were stimulated with 1×10^5^ irradiated PBMC and 18-hour ^3^H-Thymidine incorporation assays were performed (as diagrammatically shown in Figure 1; step# 2, left) and the data are shown as: **(A) CPM:** ^3^H-TdR uptake in MLR of the responder to the original stimulator used in generating the MLR-Tregs in the presence of the indicated number of modulator cells. Note that inhibition by MLR-Treg modulators is demonstrated by the differences between modulator Treg points, (right side), vs. fresh Ax (autologous irradiated PBMC) added as modulator controls (left side) (** = p<.01; n = 10). **(B) Percentage inhibition:** The CPM values (from A) were converted to percent inhibition (Tregs vs. Ax; see Methods for the formula) and allospecific vs. non-specific inhibition is shown. For allospecific inhibition the stimulators were from the original stimulators used for generating MLR-Tregs and for non-specific inhibition the stimulator PBMC were from a different totally HLA mismatched (third party) individual. Note the drastic decrease in the inhibitory effect by MLR-Tregs in the non-specific culture combinations as the modulator cell concentrations decreased (** = p<0.01; n = 5).

To test for their suppressive function, MLR-Tregs were added as third component modulators at doses of 1×10^4^, 2×10^3^ and 400 cells to freshly prepared MLRs of responders and stimulators (1×10^5^) both of which were also used in generating the MLR-Tregs. Figure 3A demonstrates that proliferation in the MLR assay was profoundly suppressed by these concentrations of modulator MLR-Tregs. This is in contrast to control assays in which fresh irradiated PBMC autologous with the original responder were tested as modulators (p<0.01).

The allospecific nature of regulation by MLR-Tregs was also assessed by using the original stimulators [used in generating the MLR-Tregs (specific)] versus totally HLA-mismatched (third party; non-specific) stimulators in the read-out MLRs. To account for the variability in the strength of the proliferative responses (CPM values) among individual experiments, the data were also expressed as percent inhibition (Figure 3B). As is shown in Figure 3B, specific inhibition was more potent than non-specific inhibition, especially with the 2 lower modulator numbers tested (p<0.01).

### Allo-specific regulation by MLR-Tregs of the micro-CML using whole PBMC responders

**Figure 4:**
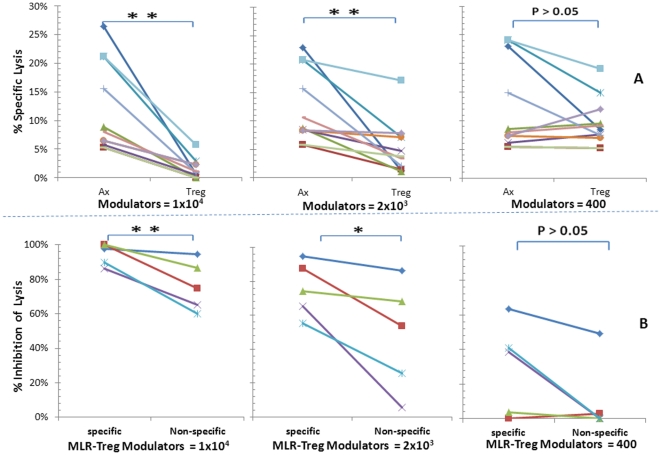
Regulatory effects of MLR-Tregs in micro-CML assays of responding whole PBMC. MLR-Tregs were added as modulators in descending concentrations of 1×10^4^, 2×10^3^ or 0.4×10^3^ cells per well to 1×10^5^ fresh responding PBMC from the same individual as the one from whom MLR-Tregs were generated. These were stimulated with 1×10^5^ irradiated allogeneic PBMC and 4-hour ^51^Cr release assays against target cells from the stimulator were performed (as diagrammatically shown in Figure 1; step# 2, middle). The data are depicted as: **(A) Percent specific lysis:** against the specific stimulator used both in MLR-Treg generation and the micro-CML readout. Similar to the data shown in Figure 3, the inhibitory effects by MLR-Tregs are depicted by the points on the right, and the absence of inhibitory effects by modulator controls (Ax) is shown by points on the left side of each graph (** = p<.01; n = 10). **(B) Percentage inhibition:** The percent specific lysis values were converted to percent inhibition (see Methods for this calculation). To test for specific inhibition, the stimulator/targets were from the original donor used for generating MLR-Tregs and for non-specific inhibition stimulator/target cells were from a third party donor. Note that allospecificity of CTL regulation is demonstrated with more significant inhibition at higher concentrations of MLR-Tregs in responses against stimulating cells from the original donor vs. those of the third party donor (** = p<0.01; * = p<0.05; n = 5).

MLR-Tregs were then added as modulators to assays using whole PBMC to generate CTL activity in the micro-CML. The CTL responses with MLR-Treg modulators were sharply reduced (inhibited), and in a dose dependent manner (Figure 4). This was compared to adding autologous irradiated responder third component control modulators which showed no inhibitory effect (Ax, Figure 4A). Inhibition occurred with both 10,000 and 2,000 modulator cells/well (p<0.01) but was not as reproducible with the lowest MLR-Treg modulator concentration (400 cells/well).

Regulation of the micro-CML by MLR-Tregs also showed allospecificity. This was demonstrated by the use of original versus third party stimulators. Inhibition was significantly stronger using the original stimulators (p<0.01 and <0.05 indicating specificity using the highest and intermediate modulator MLR-Treg concentrations respectively) (Figure 4B).

### Lack of allospecificity of CTL regulation by MLR-Tregs if purified CD8^+^ cells were used as responders

**Figure 5:**
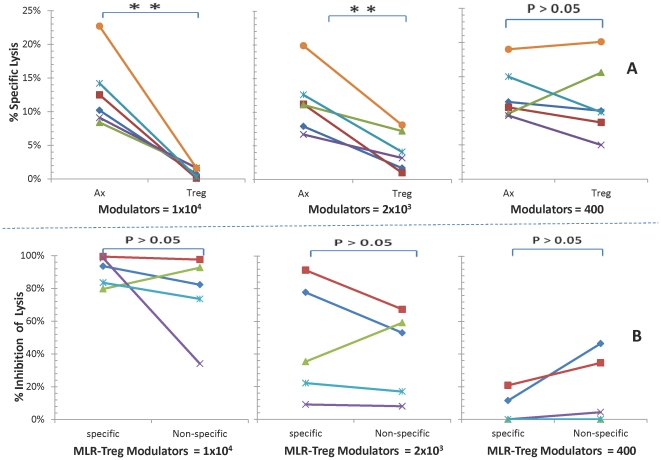
Regulatory effects of MLR-Tregs in micro-CML assays of responding purified CD8^+^ cells. Micro-CML inhibition assays were performed as described in Figure 4, except that 5×10^4^ purified CD8^+^ cells rather than whole PBMC (1×10^5^) were used as responders. The data are depicted as: **(A) Percent specific lysis:** against the specific stimulator used both in MLR-Treg generation and the micro-CML readout. Similar to the data in Figure 4A, the lysis of target cells was decreasingly inhibited by decreasing concentrations of MLR-Tregs (** = p<.01; n = 6). **(B) Percentage inhibition:** the percent specific lysis values were converted to percent inhibition. In contrast to the findings depicted in Figure 4B, inhibition of purified responding CD8^+^ CTL activity did not appear to be as clearly allospecific, i.e. there was a lack of significant differences between the points on the right side vs the left side of each graph in the lower row (p>0.05; n = 5).

It was questioned whether CD8^+^ cells purified from PBMC could be regulated by MLR-Tregs in generating CTL. Accordingly, CD8^+^ cells were immunoselected from whole blood and were tested as responders in MLR-Treg modulated cultures. The inhibition was similar to that of unpurified PBMC described above, in that the CTL activity of purified CD8^+^ cells was also inhibited by MLR-Tregs when compared to control (Ax) modulators (p<.01 at the highest and intermediate modulator concentrations) (Figure 5A). However, in contrast with assays in which non-purified PBMC were used as the responders, inhibition of CD8^+^ responders did not appear to be as prominently allospecific. In these latter experiments (Figure 5B), the degree of lysis inhibition appeared similar between allospecific and non-specific stimulating cells (the same stimulator/targets used in MLR-Treg generation vs. third party stimulator/targets) (p>0.05). These data suggest that the specificity of CTL regulation by MLR-Tregs might be due to an indirect rather than a direct effect on the CD8 responders.

### Regulatory allospecificity of CTL reactivity by MLR-Tregs requires cognate CD4^+^ T cell recognition

**Figure 6:**
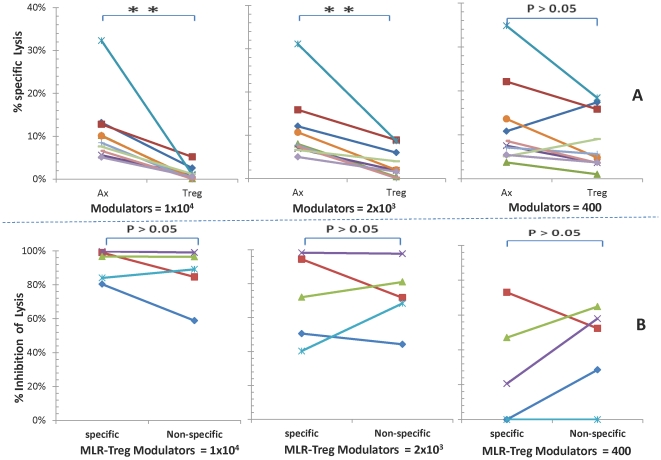
MLR-Tregs non-specifically regulate cytotoxic activity in micro-CML generated by purified CD8^+^ plus autologous non-T “APC” responders. Micro-CML inhibition assays were performed with purified CD8^+^ responders as described in Fig. 5, but in presence of non-T “APCs” autologous to the responders, and the data are shown as: **(A) Percent specific lysis:** against the specific stimulator used both in MLR-Treg generation and the micro-CML readout. The lysis of target cells was decreasingly inhibited by decreasing concentrations of modulating MLR-Tregs (points on the right side) as opposed to none seen using control (Ax) modulators (points on the left side) (** = p<.01; n = 10). **(B) Percentage inhibition:** the percent specific lysis values were converted to percent inhibition. In contrast to the findings depicted in Figure 4B, using the original vs third party stimulating cells, inhibition of CTL activity generated by purified CD8^+^ plus non-T “APC” did not appear to be allospecific. Note the lack of significant differences between the points on the right side vs the left side of each graph in the lower row (n = 5).

We then questioned whether the loss of regulatory specificity could be restored by adding back other PBMC components to the purified responder CD8^+^ cells. First, autologous “APCs” (see Methods) were added back to these purified CD8^+^ responding cells in the MLR-Treg modulated cultures (Figure 6A). However, the allospecificity of lysis inhibition could not be restored, i.e., the regulation of lysis by MLR Tregs was the same using the original stimulator/targets (specific) compared with third party (nonspecific) stimulator/targets (p>0.05) (Figure 6B).

**Figure 7:**
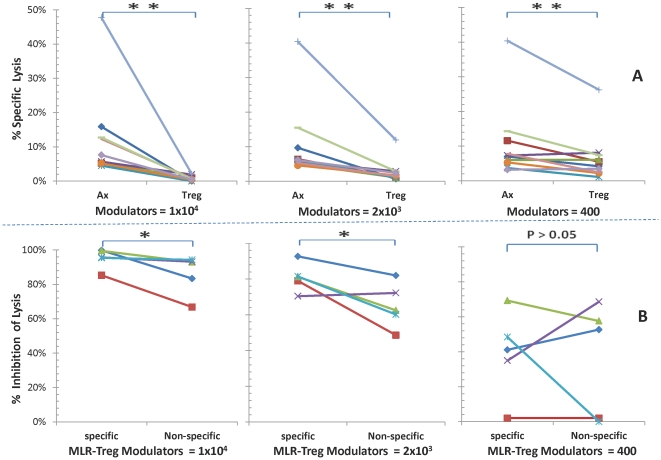
MLR-Tregs allospecifically regulate cytotoxic activity in micro-CML generated by purified CD8^+^ plus purified autologous CD4^+^ responders. Micro-CML inhibition assays were performed with purified CD8^+^ responders as described in Figure 5, but in presence of purified CD4^+^ cells autologous to the responders; the data are depicted as: **(A) Percent specific lysis:** against the specific stimulator used both in MLR-Treg generation and the micro-CML readout. The lysis of target cells was decreasingly inhibited by decreasing concentrations of modulating MLR-Tregs (points on the right side) as opposed to none seen using control modulators (points on the left side) (** = p<.01; n = 10). **(B) Percentage inhibition:** the percent specific lysis values were converted to percent inhibition. Noteworthy is that in contrast with the findings depicted in Figure 5B (but similar to those of Figure 4B), using the original vs third party stimulator/target cells, allospecific lytic inhibition was restored by using purified CD8^+^ to which purified autologous CD4^+^ cells were added (* = p<0.05; ** = p<0.01; n = 5).

In contrast, when purified autologous CD4^+^ T cells were added back to purified CD8^+^ cells, not only did inhibition of CTL activity by these MLR-Tregs occur (Figure 7A), but also the allospecificity of CTL regulation was restored. This was demonstrated when stimulator/target cells from original donors vs. third party donors were compared (p<0.05 showing differences at the highest and intermediate modulator concentrations, Figure 7B). These data indicated that purified CD4^+^ T cells appeared to play a necessary role in the regulatory specificity of CTL activity by the MLR-Tregs. As such, the MLR-Tregs required the presence of their cognate CD4^+^ T cells to restore/enhance regulation specific for the original stimulator.

### MLR-Tregs suppress the proliferation of purified CD8 cells

**Figure 8:**
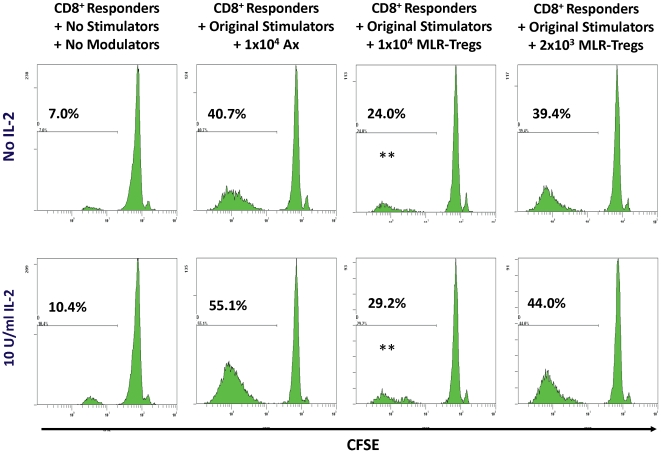
MLR-Tregs suppress purified CD8 allospecific proliferation. 5×10^4^ CFSE labeled purified CD8 cells were cultured with 1×10^5^ irradiated PBMC from the original stimulator (used in generating MLR-Tregs) together with indicated numbers of autologous MLR-Treg modulators or autologous irradiated controls (Ax), either the presence (top row) or absence (bottom row) of IL-2 (10 U/ml). After 7 days in culture flow cytometric assays were performed and the percentage of CFSE-diluted cells was estimated after gating on viable lymphocytes followed by CD8^+^ cells. It was observed that the irradiated stimulators and Ax died off by day 7 (not shown); even the few that remained were gated out on CFSE vs. CD8 density-plot during the analysis. Note the increasing percentages of (CFSE diluted) proliferating cells with decreasing concentrations of the MLR-Treg modulators. In the left column are depicted the results of negative control cultures (CD8 responders) in the absence of allogeneic stimulators or modulators. The figure is representative of 3 such experiments. (** = p<0.01; n = 3).

**Figure 9:**
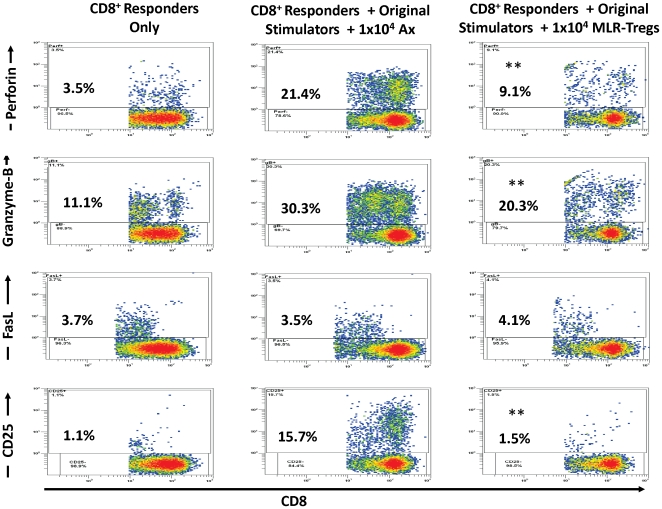
MLR-Tregs inhibit the expression of perforin, granzyme B and CD25 on responding CD8^+^ cells. 5×10^4^ PKH26 labeled purified responder CD8 cells were cultured with the original stimulators (1×10^5^) used in generating MLR-Tregs, in the presence of 1×10^4^ autologous modulator MLR-Tregs (right) vs. autologous modulator controls (Ax; middle). After 7 days in culture, the expression of intracellular Perforin-A, Granzyme-B, and membrane CD25 was assessed by flow cytometry. The CD8^+^ responder cells were gated and the PKH26^high^ non-proliferating and PKH26-diluted proliferating cells were analyzed. It was observed that the irradiated stimulators and Ax died off by day 7 (not shown); even the few that remained were gated out on CFSE vs. CD8 density-plot during the analysis. Note that there was a profound inhibition of both proliferation (PKH26 dilution) and expression of Perforin-A, Granzyme-B and CD25 by MLR-Tregs. This experiment is representative of 4 similar ones. (** = p<0.01; n = 4).

Purified CD8^+^ responders were labeled with CFSE and cultured with the original specific allogeneic stimulators and with the decreasing concentrations of allospecific MLR-Treg modulators. When followed-up in flow cytometry, these CD8^+^ cells showed a lack of CFSE dilution with the higher concentration of MLR-Treg modulators. This signified inhibition of a proliferative response (Figure 8). This was in contrast with CFSE dilution (proliferation, i.e. no inhibition) seen with the (positive) controls cocultured with similar numbers of fresh autologous irradiated cells. Thus CD8^+^ proliferation was profoundly inhibited by MLR-Tregs. Such inhibition of proliferation was also observed when PKH26 labeled CD8^+^ responders were used (Figure 9). This inhibition occurred either in the presence or absence of IL2 (Figure 8) (n = 3).

### Inhibition of CTL differentiation and activation molecules by MLR-Tregs

To further analyze the mechanism of CTL regulation mediated by MLR-Tregs, the levels of effector molecules and activation markers on CD8^+^ responders reacting to the original specific stimulator were assessed (Figure 9). Intracellular expression of the cytolytic molecules Perforin-A and Granzyme B in the proliferating CD8^+^ responder cells in flow cytometry was found to be profoundly inhibited in the presence of MLR-Tregs. Fas-ligand expression was not affected (data not shown). The expression of the (membrane) activation marker CD25 was also drastically inhibited (Figure 9).

## Discussion

It has been proposed that regulation of CTL reactivity by CD4^+^CD127^−^CD25^+^FOXP3^+^ Tregs may be a mechanism by which anti-donor responses are controlled after organ transplantation [4], [21], [22]. However, most of the recent studies performed to analyze this, utilized non-allogeneic Treg generating conditions. This has included anti-CD3/anti-CD28 antibody activation, peptide pulsed dendritic cells [23], [24], [25], [26] or exogenous addition of cytokines such as TGF-β and IL-2 [23], [24], [25], [26]. So as to more closely approximate conditions of cellular alloimmunity in human organ transplantation, we used whole PBMC in MLR to generate such Tregs. These cells had the phenotypic hallmark of being CD4^+^CD127^−^CD25^+^FOXP3^+^, defining regulatory T cells [17] (Figure 2). When purified CD4^+^CD127^−^CD25^+^FOXP3^+^ cells generated in MLR (designated MLR-Tregs) were added as modulators into a primary readout MLR, they suppressed the proliferative response in a dose dependent and allospecific manner (Figure 3B), as in our previous report [17]. The present study has been extended to analyze the regulatory effects of these MLR-Tregs on cytotoxic alloreactivity and the mechanism of this action using a micro-CML assay [20]. The lytic function of CTLs was sharply inhibited by the presence of MLR-Tregs in a dose dependent and alloantigen specific manner when whole PBMCs were used as micro-CML responders (Figure 4A). Although the majority of these cells were CD25^high^ (see Figure 1) they were not deliberately selected to be so. Although speculatively they were thymic derived ‘natural’ Tregs, because they were alloactivated, it was not considered likely that they would express the “Helios” marker found in naïve thymic Tregs [27].

Camara [14] described that human naturally occurring CD4^+^CD25^+^ regulatory T cells isolated from fresh PBMC could impair CTL activity, somewhat analogous to the present report using MLR generated CD4^+^CD127^−^CD25^+^FOXP3^+^ Tregs. However, to our knowledge, the present study shows for the first time that such MLR generated Tregs can regulate CTL reactivity with donor *allospecificity*, more cogent information in human organ transplantation. These MLR-Tregs appear to have acquired what might be termed “regulatory memory” *ex vivo*, acting with more potent suppression using the original stimulator. A recent report by Peters et. al. [28] demonstrated that human naturally occurring Tregs could be expanded *ex vivo* to acquire full antigen-specificity when stimulated by HLA mismatched irradiated PBMCs in the presence of IL-2 and IL-15. This specificity was detected by a proliferation assay of CD4^+^ cells. It was emphasized that primary allogeneic stimulation was a prerequisite. This is consistent with the present findings showing donor-specific regulation of CTL by MLR-Tregs when whole PBMC were used to generate CTLs. It is also consistent with the allospecific recruitment phenomenon caused by MLR-Tregs on autologous MLR responding cells, described in our previous report [17].

In the present study in order to explore the direct regulatory effect on CD8^+^ cells by MLR-Tregs, purified CD8^+^ cells instead of PBMCs were used as responders to generate CTLs in the presence of IL-2 (10 U/ml). Suppression of lytic activity was still observed. It was not eliminated in the presence of exogenous IL-2. This supports recent studies demonstrating that addition of exogenous IL-2 had no effect on Treg mediated suppression of mRNA production in responder T cells [14]. However, unlike the allospecific suppression of CTL reactivity by MLR-Tregs when using PBMC as responders to generate CTLs, the regulatory specificity (variably) disappeared when PBMCs responders were replaced by purified CD8^+^ cells to generate CTLs. Allospecific regulation was reconstituted by addition of CD4^+^ T cells (Figure 7B) but not by Non-T “APCs” (Figure 5). This appeared to indicate that MLR-Tregs would need (autologous) cognate responding CD4^+^ T cells present to exert their regulatory allospecificity, but that non-allospecific suppression could occur in the absence of CD4^+^ cells. In recent studies, CD4^+^ T cells have been shown to play a critical role in the CTL expansion and differentiation [29], [30], [31], [32]. In most experimental systems analyzing the effect of Tregs *in vitro*, there is a requirement for cell-cell contact for the regulatory effect occur [10], [14], [33], [34], [35]. Likewise, Tregs and effector CTLs have been observed to be in close association with each other in a number of *in vivo* or *in situ* studies [9], [36]. We have actually performed transwell diffusion chamber experiments to further pursue this point and found that the cytotoxic regulatory effect was limited to direct cell-to-cell contact between the CD4^+^CD127^−^CD25^+^FOXP3^+^ cells and the CD8 cells generating cytotoxicity. If the putative Tregs were enclosed in the upper chambers there was no regulatory effect seen in the readout CD8 cells of the lower chambers (See Table S1). Therefore, envisioning a requirement for cell-cell contact in the present system also is consistent with these studies. Although CD8^+^ cells plus “APCs” did not reconstitute the regulatory allospecificity of MLR-Tregs, it is still possible that true APCs might play a collaborative role in the regulation of CD4^+^ T cells [37], [38].

Finally, the present experiments demonstrate that MLR-Tregs can suppress CD8^+^ proliferation when stimulated by allogeneic PBMCs, and that exogenous IL-2 (10 U/ml) did not block this suppression. Moreover, the expression of the cytolytic molecules perforin and granzyme B, but not FasL, in CD8 cells was reduced, indicating possible inhibition of CD8^+^ effector functions. The expression of CD25 was decreased indicating that the activation of CD8^+^ cells was inhibited. Previous reports about these issues have been somewhat conflicting. In a mouse tumor model, activated or antigen-specific CD4^+^ Tregs did not inhibit CD8 proliferation and their differentiation to CTL, but blocked CTL killing [6], [36]. However, in a human tumor model, intra-tumor Treg cells were described inhibiting CD8^+^ proliferation and granule production [39]. In a mouse model CD4^+^CD25^+^ Tregs suppressed CD8^+^ proliferation induced by both polyclonal and Ag-specific stimuli, in which activation was also inhibited, reducing both IL-2 production and CD25 expression [40]. Similar findings were also reported in human HCV and HIV infected patients [10], [33], [41]. In humans CD4^+^CD25^+^ Tregs inhibited both CD8 proliferation and the expression of perforin and granzyme B at the transcriptional level [14]. These variable findings in diverse experimental models need further clarification.

In conclusion, human CD4^+^CD127^−^CD25^+^FOXP3^+^ regulatory T cells generated in MLR can inhibit CD8^+^ CTL lytic function both allospecifically and non-specifically. Regulatory allospecificity appears to require the presence of cognate CD4^+^ T cells. CTL regulation appears to be mediated through impaired proliferation, inhibited expression of the cytolytic molecules perforin and granzyme B, and decreased CD25 expression. Speculatively, our findings add support to the notion of utilizing such *ex vivo* generated Tregs in clinical organ and tissue transplantation.

## Supporting Information

Table S1 Assessment of cell-cell contact requirement for CTL inhibitory activity by MLR-Tregs in the micro-CML*.
(DOC)
