# Supplementary material for: Requirement of Cognate CD4+ T-Cell Recognition for the Regulation of Allospecific CTL by Human CD4+CD127−CD25+FOXP3+ Cells Generated in MLR
Source: PLoS One. 2011 Jul 22;6(7):e22450. doi: 10.1371/journal.pone.0022450 (PMC3142165; doi:10.1371/journal.pone.0022450)
Supplement: Table S1 — Assessment of cell-cell contact requirement for CTL inhibitory activity by MLR-Tregs in the micro-CML*. (DOC) [file pone.0022450.s001.doc]

| Table S1: Assessment of cell-cell contact requirement for CTL inhibitory activity by MLR-Tregs in the micro-CML* | | | | |
| --- | --- | --- | --- | --- |
|  | *Modulator Cells* | *1x10^5^* | *2x10^4^* | *4x10^3^* |
|  | Ax | 27.8 | 27.8 | 28 |
| Regular Cultures | MLR-Tregs | 12.5 | 20.4 | 27.5 |
|  | **(% Inhibition by MLR-Tregs)** | **(55.0%) **** | **(26.6%)**** | **(1.8%)** |
|  | Ax | 21.5 | 19.4 | 16.1 |
| Transwell Cultures | MLR-Tregs | 23.4 | 19.2 | 23.5 |
|  | **(% Inhibition by MLR-Tregs)** | **(0.0%)** | **(1.0%)** | **(0.0%)** |
| * 1x10^6^ Responder PBMC were stimulated with 1x10^6^ irradiated PBMC in presence of indicated third component modulators. The modulators were placed either in regular 24 well plates (Regular Cultures) or were kept separated from the responders and stimulators in the top transwells (Transwell Cultures). After 7 days 4-hour ^51^Chromium release assays were performed against targets from the specific stimulator and the data were calculated as % specific lysis and percentage inhibition by MLR-Tregs.  ** p<0.05. | | | | |
